# Experimental Design (2^4^) to Improve the Reaction Conditions of Non-Segmented Poly(ester-urethanes) (PEUs) Derived from α,ω-Hydroxy Telechelic Poly(ε-caprolactone) (HOPCLOH)

**DOI:** 10.3390/polym17050668

**Published:** 2025-02-28

**Authors:** Jaime Maldonado-Estudillo, Rodrigo Navarro Crespo, Ángel Marcos-Fernández, María Dolores de Dios Caputto, Gustavo Cruz-Jiménez, José E. Báez

**Affiliations:** 1 Departament of Chemistry, University of Guanajuato (UG), Noria Alta S/N, Guanajuato 36050, Mexico; 2 Instituto de Ciencia y Tecnología de Polímeros (ICTP), Consejo Superior de Investigaciones Científicas (CSIC), Juan de la Cierva 3, 28006 Madrid, Spainamarcos@ictp.csic.es (Á.M.-F.); mariad.dedios@ictp.csic.es (M.D.d.D.C.); 3 Departament of Pharmacy, University of Guanajuato (UG), Noria Alta S/N, Guanajuato 36050, Mexico

**Keywords:** experimental design, poly(ester-urethanes), number-average molecular weight, poly(ε-caprolactone), telechelic

## Abstract

Aliphatic unsegmented polyurethanes (PUs) have garnered relatively limited attention in the literature, despite their valuable properties such as UV resistance and biocompatibility, making them suitable for biomedical applications. This study focuses on synthesizing poly(ester-urethanes) (PEUs) using 1,6-hexamethylene diisocyanate and the macrodiol α,ω-hydroxy telechelic poly(ε-caprolactone) (HOPCLOH). To optimize the synthesis, a statistical experimental design approach was employed, a methodology not commonly utilized in polymer science. The influence of reaction temperature, time, reagent concentrations, and solvent type on the resulting PEUs was investigated. Characterization techniques included FT-IR, ^1^H NMR, differential scanning calorimetry (DSC), gel permeation chromatography (GPC), optical microscopy, and mechanical testing. The results demonstrated that all factors significantly impacted the number-average molecular weight (*M*_n_) as determined by GPC. Furthermore, the statistical design revealed crucial interaction effects between factors, such as a dependence between reaction time and temperature. For example, a fixed reaction time of 1 h, with the temperature varying from 50 °C to 61 °C, did not significantly alter *M*_n_. Better reaction conditions yielded high *M*_n_ (average: 162,000 g/mol), desirable mechanical properties (elongation at break > 1000%), low levels of unreacted HOPCLOH in the PEU films (OH/ESTER response = 0.0008), and reduced crystallinity (Δ*H*_m_ = 11 J/g) in the soft segment, as observed by DSC and optical microscopy. In contrast, suboptimal conditions resulted in low *M*_n_, brittle materials with unmeasurable mechanical properties, high crystallinity, and significant amounts of residual HOPCLOH. The best experimental conditions were 61 °C, 0.176 molal, 8 h, and chloroform as the solvent (ε = 4.8).

## 1. Introduction

Polyurethanes (PUs) are extensively used across a wide range of industries due to their excellent mechanical properties, chemical resistance, and the versatility of their synthesis. Conventionally, PUs are synthesized from two key components: (1) a diisocyanate (O=C=N-R-N=C=O) and (2) a macrodiol (HOROH). The use of a catalyst is optional but can enhance the reaction efficiency [1]. PUs find applications in diverse fields, including adhesives [2,3], foams [4,5], coatings [6,7,8,9], and medical devices [10,11,12].

For biomedical applications, aliphatic diisocyanates such as 1,6-hexamethylene diisocyanate (HDI), isophorone diisocyanate (IPDI), and trans-1,4-cyclohexane diisocyanate (CHDI) are often preferred. This preference stems from the fact that PUs derived from aliphatic diisocyanates decompose into less toxic byproducts compared to those synthesized from aromatic diisocyanates [13,14,15,16]. Additionally, aliphatic PUs exhibit superior resistance to ultraviolet (UV) radiation compared to their aromatic counterparts. This improvement is attributed to the absence of UV-absorbing aromatic rings in their structure [17,18], making aliphatic PUs more suitable for applications requiring prolonged exposure to sunlight.

The second essential component for PU synthesis, the macrodiol, often comprises linear or branched aliphatic polyesters, specifically α,ω-hydroxy telechelic polyesters synthesized via ring-opening polymerization. Examples of such polyesters include poly(3-hydroxybutyrate) (P3HB) [19,20] and polylactic acid (PLA) [21,22].

Another notable example of a macrodiol utilized in PU synthesis is α,ω-hydroxy poly(ε-caprolactone) (HOPCLOH), a biodegradable oligoester [23,24]. The combination of HDI and macrodiols derived from aliphatic polyesters leads to the formation of aliphatic poly(ester-urethanes) (PEUs). The synthesis of non-segmented PEUs derived from HOPCLOH and aliphatic diisocyanates presents opportunities to investigate the relationship between their structure and properties [25,26]. Despite the significance of this class of aliphatic PEUs and their potential applications, research in this area remains limited. In contrast, a significant body of work has focused on PUs synthesized from aromatic diisocyanates [27,28,29,30,31].

A critical parameter in polymer synthesis and the resulting properties is the number-average molecular weight (*M*_n_), as many physical characteristics of polymers are directly influenced by this value. Notably, for PEUs derived from PCL and HDI, *M*_n_ is often not reported in the literature. This omission is partly attributed to the cross-linked nature or poor solubility of some of these polymers [23,32,33,34,35,36].

Our group previously conducted a systematic study on the influence of the molar ratio of reactants, commonly referred to as the isocyanate index (HDI/HOPCLOH), on polymer properties [37]. The isocyanate index is a key determinant of *M*_n_, cross-linking density, and chain branching in the resulting PEUs. Indices greater than 1 typically promote higher cross-linking densities or increased branching [36,38,39,40,41], while indices below 1 often result in incomplete polymerization. This incomplete reaction leads to low *M*_n_ values and little to no cross-linking or branching [42,43,44], ultimately producing PEUs with poor mechanical properties. Therefore, understanding and optimizing the diisocyanate index is essential for improving the performance of these materials.

Another critical factor influencing polymerization is reaction temperature. Proper control of the temperature can significantly impact the outcome of the process. For instance, some polyurethanes can be synthesized at ambient temperature [45,46]. It is well established that increasing the reaction temperature accelerates reaction kinetics in PU synthesis [47]. However, higher temperatures also increase the likelihood of side reactions, such as allophanate cross-linking or branching [48], which can alter the structural and mechanical properties of the polymer. Conversely, insufficient reaction temperatures [49] may result in low *M*_n_ values due to incomplete reactions. Thus, the reaction temperature must be carefully optimized to balance reaction kinetics with the structural integrity of the synthesized polymer [38,50,51].

The choice of solvent is another significant factor, as it can influence the reaction mechanism [52,53,54,55] and its ability to dissolve both the reactants and the growing polymer chains [31,56,57]. Adequate solubility is critical for preventing premature gelation during the reaction process. To mitigate the risk of gelation or excessive viscosity during polymerization, it is common practice to introduce a suitable quantity of solvent when an increase in viscosity is observed [39,58,59].

Time and concentration are two critical parameters that significantly influence the kinetics of polymer synthesis [60,61]. Concentration, in particular, plays a pivotal role by increasing the frequency of molecular collisions, thereby enhancing both catalytic effects (such as favoring the diffusion of a metal catalyst) and the autocatalytic activity of urethane groups [62,63]. To synthesize PEUs with the desired properties, it is essential to appropriately adjust these and other factors in the reaction process. Several methodological approaches can be employed to optimize reaction conditions and achieve specific or multiple desired outcomes.

The trial-and-error method is a traditional approach that involves systematically refining reaction conditions based on prior results. While this method offers flexibility and allows for continuous improvement, it can be time-consuming and inefficient, often leading to local rather than global optimization [64].

In contrast, machine learning (ML) experimentation is an advanced and more powerful approach. ML models can simulate polymerization processes, predict the effects of reaction parameters, and explore larger reaction spaces, thereby increasing the likelihood of identifying optimal conditions [65]. However, ML approaches require substantial computational resources and expertise in data modeling. Additionally, challenges such as overfitting—where the model becomes overly tailored to the training data and performs poorly on new datasets [65,66]—can reduce the reliability and generalizability of these predictions.

Among the various alternatives for experimental design, 2^n^ factorial designs are a classic and practical option. This method offers a systematic approach to improving polymerization reaction conditions and provides a more efficient exploration of the reaction space compared to iterative experimentation [67,68]. While less complex and resource-intensive than machine learning-based approaches, the 2^n^ factorial design remains highly effective in identifying critical factors.

A key advantage of the 2^n^ factorial design is its ability to evaluate multiple factors and their interactions in a structured and straightforward manner, making it an excellent starting point for optimizing reaction parameters [69]. Additionally, it is often advisable to begin with a linear mathematical model, as it is simpler and more interpretable compared to more complex techniques, such as response surface models or machine learning models. The linear nature of the 2^n^ factorial design provides a solid foundation for identifying significant factors and interactions, which can later inform the development of more advanced optimization strategies. Notably, the 2^n^ factorial design is highly adaptable and can be extended to create response surface models or serve as a basis for training machine learning models [70], effectively bridging the gap between simplicity and advanced optimization techniques.

In this study, a preliminary investigation was conducted to explore the factors influencing the synthesis of PEUs. Specifically, the study examined (1) starting material concentrations (HOPCLOH and HDI), (2) reaction temperature, (3) reaction time, and (4) solvent type. A 2^4^ factorial design (two levels per factor, with four factors) was employed to systematically investigate and optimize the reaction conditions. This approach allowed for a deeper understanding of how these factors influence the number-average molecular weight (*M*_n_), whether high or low. The factorial design enabled the identification of both the main effects of each factor and their interactions, offering a structured framework for optimizing polymerization conditions and guiding future studies.

## 2. Experiment

### 2.1. Materials

The materials utilized in this study were sourced from Sigma-Aldrich (now Merck, Darmstadt, Germany) and were characterized by their respective high purity levels: ε-caprolactone (CL) with a purity of 97%, 1,8-octanediol at 98%, ammonium heptamolybdate tetrahydrate ((NH_4_)_6_Mo_7_O_24_⋅4H_2_O) at 99%, 1,6-hexamethylene diisocyanate (HDI) at 99.0%, and two anhydrous solvents: (a) chloroform (dielectric constant 4.8) at ≥99%, containing amylenes as a stabilizer, and (b) acetonitrile (dielectric constant 38) at 99.8%. The glassware was dried in an oven at 100 °C overnight before use. Polymerization reactions were conducted using an IKA^®^ RET basic magnetic stirrer hot plate with a metal flask carrier (100 mL) and a flask inlay (25 mL), operating at a voltage of 115 V.

### 2.2. Preparation of α,ω-Hydroxy Telechelic Poly(ε-caprolactone) (HOPCLOH)

The synthesis of α,ω-hydroxy telechelic poly(ε-caprolactone) (HOPCLOH) was carried out via bulk polymerization. In a typical reaction, using a dry round-bottom flask (250 mL), the following substances were added: (a) ε-caprolactone (CL) (126.7 g, 1.35 mol) as the monomer, (b) 1,8-octanediol (15.0 g, 0.136 mol) as the initiator, and (c) ammonium decamolybdate, synthesized in situ from ammonium heptamolybdate (0.084 g), which functioned as the catalyst. After 1 h, the oligomeric macrodiol HOPCLOH was obtained in the form of a waxy substance. The resulting product exhibited a number-average molecular weight (*M*_n_), as determined by ^1^H NMR, of 1177 g/mol. HOPCLOH: ^1^H NMR (500 MHz, CDCl_3_, ppm): δ 4.04 (*t*, [-CH_2_-O-], PCL and *t*, [-CH_2_-O-CO], Oct), 3.64 (*t*, 4H, [(-CH_2_-OH)_2_], PCL and [(-CH_2_-OH)_2_], Oct), 2.31 (*t*, 2H, (-CH_2_-CO-O-), PCL), 1.64 (*m*, 4H, [(CH_2_)_2_], PCL), 1.58 (*q*, 4H, [(CH_2_)_2_], Oct), 1.36 (*t*, 2H, [CH_2_], PCL), 1.32 (*s*, 8H, [CH_2_]_4_, Oct). ^13^C NMR (125 MHz CDCl_3_, ppm): δ 173.65 [-O-CO-(CH_2_)_5_-OH] PCL hydroxyl terminal group, 173.45 [-O-CO-(CH_2_)_5_-] PCL, 64.31 [-*C*H_2_-O-CO-] oct, 64.01 [-*C*H_2_-O-CO-] PCL, 62.37 [-O-CO-(CH_2_)_4_-*C*H_2_-OH, ester] PCL, 34.12 [-O-CO-*C*H_2_-(CH_2_)_4_-OH, hydroxyl terminal group] PCL, 34.01 [-*C*H_2_-CO-O-, ester] PCL, 32.18 [-O-CO-(CH_2_)_3_-*C*H_2_-CH_2_-OH, ester]. IR (cm^−1^): 3459 (*ν*, O-H, hydroxyl), 2936 (*ν*_as_, CH_2_, PCL), 2864 (*ν*_s_, CH_2_, PCL), 1722 (*ν*, C=O, PCL), 1164 (*ν*_as_, C-(C=O)-O, PCL), 731 (*ρ*, CH_2_, PCL). DSC: Δ*H*_m_ = 85 J/g, *T*_m1_ = 39 °C, *T*_m2_ = 43 °C *x_i_ (%)* = 63%.

### 2.3. Synthesis of Poly(ester-urethanes) (PEUs) from HOPCLOH

**Scheme 1:**
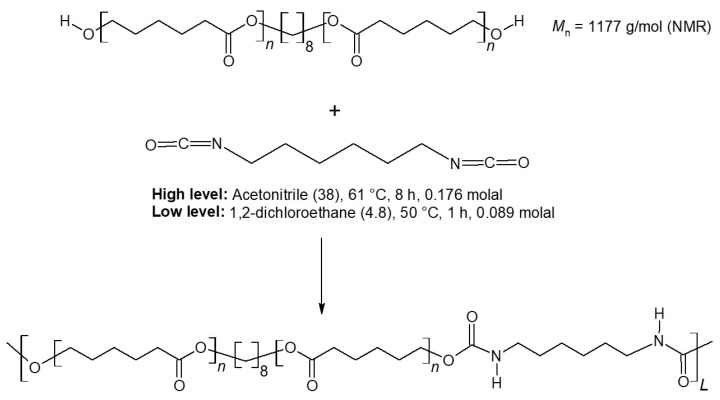
Reagents and reaction conditions for the synthesis of PEUs to improve the process.

The initial conditions for the synthesis of poly(ester-urethanes) (PEUs) were outlined in previous studies [23,71]. The molar ratio of 1,6-hexamethylene diisocyanate (HDI) to macrodiol (HOPCLOH) was set at 1.14:1. The experiments were conducted in this way (PEUs). The specific conditions were as follows: 0.2960 g (0.00176 mol) of HDI and 1.991 g (0.00154 mol) of HOPCLOH, which had a theoretical degree of polymerization (DP) of 10 [experimentally, *M*_n_(NMR) = 1177 g/mol, as determined by ^1^H NMR, considering 8% unreacted diol (1,8-octanediol) in the original HOPCLOH], were introduced into a 25 mL round-bottom flask, previously dried in an oven overnight. The polymerization reaction was performed using chloroform or acetonitrile as the solvent at concentrations of 0.089 or 0.176 molal. The reaction was conducted at 50 °C or 61 °C for 1 h or 8 h (see Scheme 1). Following the polymerization process, the resulting PEU solution was transferred onto a Teflon plate bordered by a glass ring, and the solvent was allowed to evaporate over a period of 12 h or until it had fully evaporated. PEU-20: ^1^H NMR (500 MHz, CDCl_3_, ppm): *δ* 4.73 (*s*, ^1^H, [NH], urethane), 4.05 (*t*, 2H, [CH_2_O], PCL and *t*, 2H, [CH_2_O], Oct), PCL and [(-CH_2_-OH)_2_], Oct), 3.63 (*t*, 4H, [(-CH_2_-OH)_2_], PCL and [(-CH_2_-OH)_2_], Oct), 3.14 (*t*, 4H, [(-CH_2_-NH-CO-O-)_2_], urethane), 2.31 (*t*, 2H, (-CH_2_-CO-O-), PCL), 1.64 (*m*, 4H, [(CH_2_)_2_], PCL and *q*, 4H, [CH_2_)_2_], Oct), 1.47 (*q*, 4H, [(CH_2_)_2_], urethane), (1.37 (*q*, 2H, [CH_2_], PCL, and *q*, 4H, [CH_2_]_2_, urethane), 1.32 (*s*, 8H, [CH_2_]_4_, Oct. ^13^C NMR (125 MHz, CDCl_3_, ppm): IR (cm^−1^): 3339 (*ν*, N–H, urethane), 2937 (*ν*_as_, CH_2_, PCL), 2858 (*ν*_s_, CH_2_, PCL), 1720 (*ν*, free C=O, PCL), 1681 (*ν*, C=O, overlapping hydrogen-bonded urethane.) 1625 cm^−1^: ν(N–H) (urea) 1536 (*δ*, N–H, urethane), 1465 (*δ*_s_, CH_2_, PCL), 1190 (*ν*_as_, C-(C=O)-O, PCL), 1043 (*ν*_as_, O-C-C, PCL), 732 (*ρ*, CH_2_, PCL). DSC (soft segment (PCL)): Δ*H*_m_ = 31 J/g, *T*_m_ = 43 °C, *x*_i_ (%) = 23%.

### 2.4. Experimental Design

All statistical analyses were conducted using the Minitab 19^®^ software [72], with a significance threshold of α = 0.05 applied to all statistical tests [69]. To optimize the reaction conditions for maximizing the number-average molecular weight (*M*_n_) of the PEUs, a comprehensive list of factors potentially influencing *M*_n_ was developed. A 2^n^ factorial experimental design was employed, where the number 2 indicated two levels in each factor, and n represented the number of factors. Consequently, the current experimental design used four factors: (1) type of solvent, (2) temperature, (3) reaction time, and (4) concentration of reagents (Table 1). To visualize the experiments in the 2^4^ experimental design, all 16 treatments are presented in Table S1, where each treatment was duplicated, resulting in a total of 32 experimental runs. Additional factors that might affect *M*_n_ were held constant to control variability. The sequence of these runs was randomized to reduce the risk of systematic bias. During each run, relative environmental humidity (expressed as a percentage) was monitored to determine whether a positive correlation existed between this covariate and *M*_n_.

## 3. Measurements

### 3.1. Fourier Transform Infrared Spectroscopy (FT-IR)

Infrared spectra were obtained using the attenuated total reflectance (ATR) technique with a Perkin-Elmer Spectrum One FT-IR spectrometer and a Perkin-Elmer Spectrum Two FT-IR spectrometer.

### 3.2. Nuclear Magnetic Resonance (NMR)

^1^H and ^13^C NMR spectra were recorded at room temperature using a Bruker Avance III HD 500 MHz spectrometer, with CDCl_3_ as the solvent. Chemical shifts (δ) are reported in parts per million (ppm) and were referenced to the residual solvent signal: ^13^C at δ = 77.16 ppm and ^1^H at δ = 7.26 ppm.

### 3.3. Gel Permeation Chromatography (GPC)

For PEUs, a PerkinElmer gel permeation chromatograph (200 Series LC pump) equipped with a refractive index detector (IR 200a) was used. ResiPore columns (Polymer Laboratories, Long Beach, CA, USA) were conditioned at 70 °C, with a flow rate of 0.3 mL/min using HPLC-grade dimethylformamide (DMF) containing 0.1 wt% LiBr as the mobile phase. Calibration was performed using polystyrene standards (Polymer Laboratories).

### 3.4. Mechanical Properties

Tensile measurements were conducted using a Zwick/Roell Z005 instrument (Zwick/Roell, Singapore) equipped with a 500 N load cell. The tensile tests were performed at a test speed of 200 mm/min. Type 3 dumbbell-shaped test pieces (according to ISO 37) were cut from the films.

### 3.5. Differential Scanning Calorimetry (DSC)

Thermograms were obtained using a DSC Q200 instrument. The experiments consisted of several cycles: (1) heating from 25 °C to 100 °C, (2) isothermal hold for 1 min at 100 °C, (3) cooling from 100 °C to −30 °C, (4) isothermal hold for 1 min at −30 °C, and (5) final heating from −30 °C to 100 °C. The entire cycle was performed at a heating rate of 10 °C/min. The melting temperature and enthalpy of fusion (Δ*H*_m_) were determined from the peak of the endothermic transition during the second heating cycle.

The degree of crystallinity of PCL was calculated using the following formula:

x_e_ = ∆*H*_e_/∆*H*_e_^0^

where x_e_ is the degree of crystallinity, Δ*H*_e_ was obtained from the area under the thermal transition curve of the soft segment and PCL transition, and Δ*H*_e_^0^ corresponds to the enthalpy of fusion for perfect PCL crystals (135.3 J/g, as reported by Crescenzi [73]).

### 3.6. Polarized Optical Microscopy (POM)

POM micrographs were acquired using a NIKON optical microscope (MODEL). Photographs were captured using an iPhone 13. Samples were prepared on glass slides as thin films melted at 120 °C using a hot plate, applying manual pressure between two glass slides containing the sample and a coverslip. The samples underwent an isothermal treatment at 50 °C for 24 h and were subsequently cooled to room temperature before analysis. All micrographs were collected at 40× magnification.

## 4. Results and Discussion

### 4.1. Preparation and Characterization of α,ω-Hydroxy Telechelic Poly(ε-caprolactone) (HOPCLOH)

**Figure 1:**
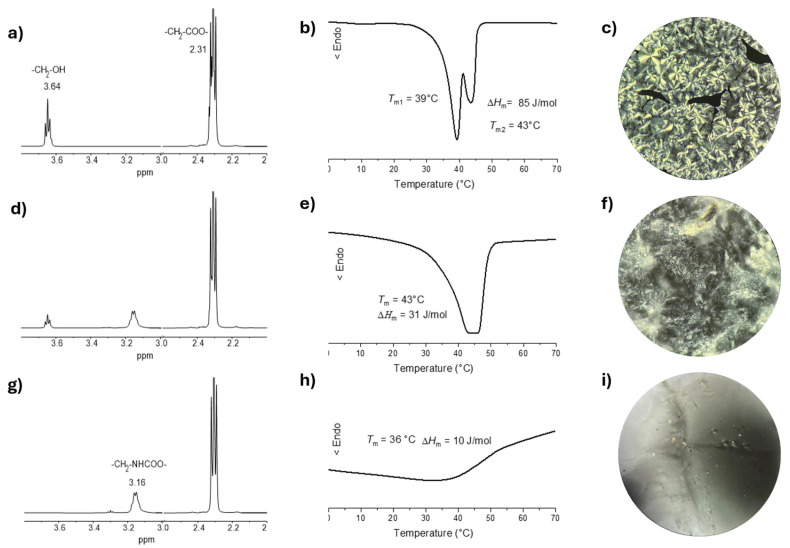
From left to right, ^1^H NMR spectra, differential scanning calorimetry (DSC) thermograms, and optical microscopy images are displayed. Part (**a**) shows the ^1^H NMR spectrum of the HOPCLOH precursor, part (**d**) shows the spectrum of a PEU-20 (*M*_n_ = 7500 g/mol) obtained with acetonitrile under the worst experimental conditions, and part (**g**) shows the spectrum of a PEU-13 (*M*_n_ = 171,000 g/mol) obtained with chloroform under the best experimental conditions. The following column presents the corresponding DSC thermograms: (**b**) HOPCLOH precursor, (**e**) PEU synthesized under the worst conditions, and (**h**) PEU synthesized under the best conditions. The optical micrographs are in (**c**,**f**,**i**) from HOPCLOH, PEU-20, and PEU-13, respectively.

The precursors of poly(ester-urethanes) (PEUs) typically consist of a macrodiol and a diisocyanate [23,26]. In this study, the macrodiol used was α,ω-hydroxy telechelic poly(ε-caprolactone) (HOPCLOH), which is generally synthesized via the ring-opening polymerization (ROP) of ε-caprolactone (CL). For this purpose, ammonium decamolybdate was employed as the catalyst, while 1,8-octanediol (HOROH) served as the initiator in the ROP of CL (Scheme S1). This synthetic route, using alcohol and diols as initiators, has been previously reported by our group [25,71,74]. In ROP, the degree of polymerization (DP) is controlled by the CL/HOROH molar ratio, a critical parameter since the starting material (HOPCLOH) in PEU synthesis requires a low number-average molecular weight (*M*_n_), typically in the oligomeric range. Accordingly, the HOPCLOH prepared in this study was designed with a theoretical DP of 10. After 1 h at 150 °C, the reaction reached completion. To confirm the chemical structure of HOPCLOH, the resulting oligomer was analyzed using various analytical techniques. The ^1^H NMR spectrum (Figure 1a) of HOPCLOH revealed two distinct and significant signals: one at 3.64 ppm, corresponding to the methylene group attached to hydroxyl terminals (-CH_2_-OH), and another at 2.31 ppm, representing the α-methylene group adjacent to the ester moiety [-CH_2_-(C=O)-O-]. These signals confirm the presence of α,ω-hydroxy telechelic terminal groups and the repetitive unit within the oligomer, respectively. The thermal properties of HOPCLOH were evaluated using differential scanning calorimetry (DSC). The thermogram (Figure 1b) displayed a prominent endothermic transition attributed to double melting temperatures (*T*_m_) at 39 and 43 °C. This profile is characteristic of crystallites of varying sizes in HOPCLOH [25,26]. Additionally, polarized optical microscopy (POM) confirmed the presence of semicrystalline domains, as evidenced by the formation of spherulites (Figure 1c).

The second reaction in this study involves two components: HOPCLOH and 1,6-hexamethylene diisocyanate (HDI), which serve as the starting materials for PEU synthesis (Scheme 1). The progress of the polymerization reaction between HOPCLOH and HDI was monitored using ^1^H NMR spectroscopy (Figure 1d,g). During the reaction, the signal corresponding to the ester repetitive unit [-CH_2_-(C=O)-O-] remained unchanged, while the intensity of the hydroxyl signal (-CH_2_-OH) decreased. This decrease indicates the consumption of hydroxyl groups (-OH) as they react with diisocyanate groups (-N=C=O) to form urethane linkages [-NH-(C=O)-O-]. The reaction progression was quantitatively analyzed by comparing the integral ratios of the hydroxyl and ester peaks (OH/ESTER response), referred to as the first response.

Figure 1d shows the peak assignments for poly(ester-urethane) (PEU-20, Table S2) with a magnified view of a key region in the ^1^H NMR spectrum. PEU-20 was synthesized under the least favorable conditions [50 °C, 1 h, acetonitrile (ε = 38), 0.089 molal concentration]. These conditions were deemed suboptimal based on the presence of unreacted HOPCLOH, identified by the characteristic triplet signal of the hydroxyl end group (-*CH*_2_-OH) at 3.64 ppm, and PEU-20 exhibited the highest integral value for this signal among all 32 samples. In contrast, the ^1^H NMR spectrum of the PEU-13 sample (Figure 1g) showed the absence of unreacted HOPCLOH, indicating optimal reaction conditions [61 °C, 8 h, chloroform (ε = 4.8), 0.176 molal concentration]. Additionally, the appearance of a signal at approximately 3.16 ppm (-*CH*_2_-NH-(C=O)-O-) confirmed the formation of the urethane group. The progression of the reaction was assessed via several integral ratios: the ratio of the hydroxyl peak (-*CH*_2_-OH, corresponding to unreacted hydroxyl groups) to the ester peak (-*CH*_2_-COO) was termed the HO/ESTER response, while the ratio of the hydroxyl peak (-*CH*_2_-OH) to the urethane peak (-*CH*_2_-NHCOO-) was designated as the HO/URET response, also called the second response.

Thermal analysis using DSC thermograms for both PEU-20 (Figure 1e) and PEU-13 (Figure 1h) revealed two distinct crystalline domains. For PEU-20, the enthalpy of fusion (Δ*H*_m_) decreased significantly to 31 J/mol (Figure 1e) compared to the starting material HOPCLOH (Δ*H*_m_ = 85 J/mol, Figure 1b). This reduction indicated a disruption in the crystalline domain of the PCL soft segment within the PEU, attributed to the formation of urethane groups with intermolecular hydrogen bonding, which promoted the formation of amorphous domains [25]. This disruption was further supported by the polarized optical microscopy (POM) micrograph of PEU-20 (Figure 1f), which showed a significant reduction in spherulite formation, consistent with the DSC results. For PEU-13, DSC thermograms (Figure 1h) displayed a further decrease in Δ*H*_m_ to 10 J/mol, while POM analysis (Figure 1i) confirmed the absence of spherulite structures. These results suggest an even greater disruption in the crystalline domains and a predominance of amorphous regions under the optimized reaction conditions.

**Figure 2:**
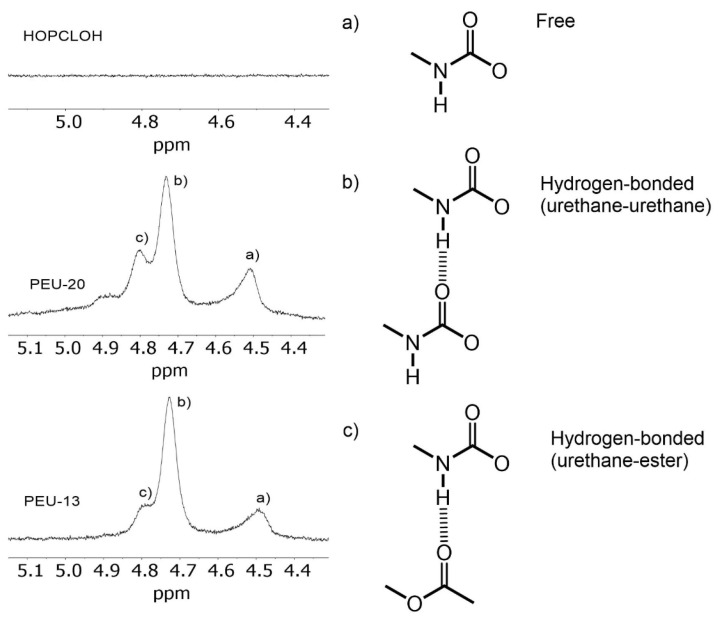
The ^1^H NMR spectra are shown in the chemical shift region of the hydrogen in the urethane group [-O-(C=O)-N-H, singlet] for (**a**) the precursor (HOPCLOH), (**b**) PEU-20 (worst conditions), and (**c**) PEU-13 (best conditions).

Complementary to this, the ^1^H NMR analysis shows another peak at 4.73 ppm, indicative of the hydrogen attached to the urethane group (N-H). Near the N-H peak, two additional less intense peaks are observed, corresponding to hydrogens from N-H groups. These peaks suggest the presence of hydrogen bonded to nitrogen in at least three distinct chemical environments (Figure 2). The peak at 4.50 ppm is likely attributed to urea hydrogen, as this value closely matches previously reported chemical shifts for *N-H* hydrogen in *N,N’-dihexylurea* [75]. The integral values of these hydrogen signals vary depending on the degree of polymerization.

**Figure 3:**
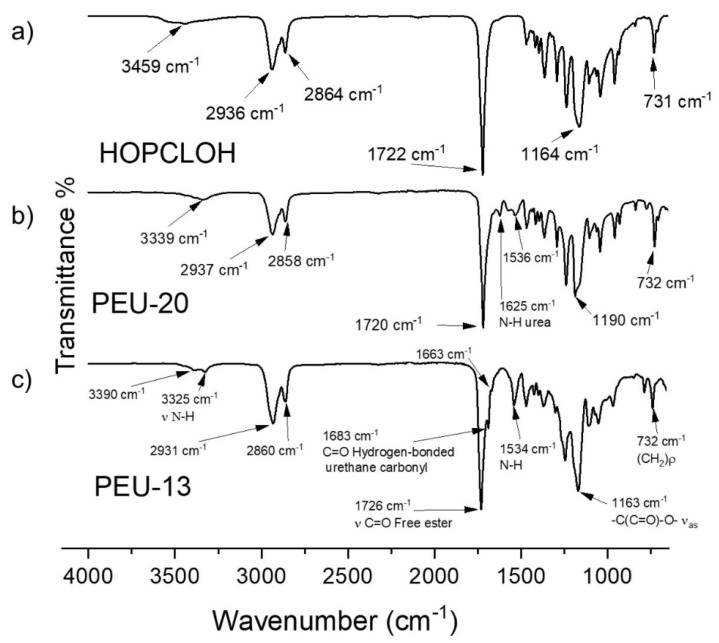
Three FT-IR spectra are shown: (**a**) the precursor HOPCLOH, (**b**) the PEU-13 with the highest *M*_n_, and (**c**) the PEU-20 sample with the lowest *M*_n_.

FT-IR analysis was performed to identify the functional groups of three different species previously characterized by ^1^H NMR, DSC, and POM (Figure 1). Figure 3a presents the expected bands in the infrared spectrum of HOPCLOH. The primary bands include one at 1723 cm^−1^, corresponding to the stretching of the ester carbonyl group, and another at 3446 cm^−1^ attributed to the hydroxyl (–OH) group involved in hydrogen bonding.

In the spectrum of PEU-20 (low *M*_n_; Figure 3b), two prominent bands are observed at 1720 cm^−1^ and 1625 cm^−1^. The latter is attributed to the formation of urea (N-H bending) [76]. Notably, the band at 1625 cm^−1^ was also detected in a sample of HDI intentionally exposed to an excess of water, which promoted the formation of polyurea (Figure S1).

Figure 3c shows the infrared spectrum of PEU-13 (high *M*_n_). In the region of the carbonyl groups, multiple bands are present. The first band, at 1726 cm^−1^, corresponds to the ester group within the repetitive unit. The second band, at 1683 cm^−1^, is traditionally attributed to the carbonyl group of urethane involved in hydrogen bonding with N-H. However, recent studies suggest that this band at 1683 cm^−1^ may also indicate the formation of branching or cross-linking in PEU [77]. The third band, at 1626 cm^−1^, appears as a slight shoulder, possibly indicating a minor presence of urea in the PEU, consistent with the urea spectrum (Figure S1). Finally, in the region of N-H stretching, two bands are observed at 3390 cm^−1^ and 3325 cm^−1^, further confirming the presence of urethane and urea functionalities.

### 4.2. 2^4^ Experimental Design: Effect of Concentration, Temperature, Solvent, and Reaction Time on the M_n_ of PEUs

A detailed investigation was conducted to evaluate the effects of four key factors—concentration (*c*), temperature (*T*), solvent (*s*), and reaction time (*t*)—on the molecular weight (*M*_n_) of poly(ester-urethanes) (PEUs). These factors were selected based on insights from the literature and prior experience, as they are believed to exert the greatest influence on *M*_n_. Each factor was analyzed at two levels: high and low (Table 1). Preliminary testing revealed that the reaction could proceed in the absence of the Sn(Oct)_2_ catalyst, which is notable. The exclusion of this catalyst is important because there had been concerns that inaccuracies in measuring the catalyst amount could introduce substantial experimental error, potentially masking real differences between the factor levels.

A 2^4^ factorial design was implemented to evaluate a total of 15 effects (Equation (S1)), including the individual impact of each factor and the interaction effects among the four factors (*c*, *T*, *s*, *t*). The analysis revealed that all four primary factors had a statistically significant influence on the molecular weight (*M*_n_), as reflected in the *M*_n_ response. The detailed findings of these significant effects were examined in the first analysis of variance (ANOVA).

Initially, the statistical model included all 15 effects (Equation (S1)). However, following the first ANOVA, it was determined that only eight of these effects were statistically significant (Table 2 and Equation (S2), vide supra). The analysis confirmed that each of the four main factors had a significant effect on *M*_n_, as supported by the ANOVA results presented in Table 2. Additionally, the presence of three significant two-factor interactions and one significant three-factor interaction indicates that *M*_n_ is influenced not only by the individual factors, but also by the specific combinations of factor levels in these interactions.

### 4.3. Analysis of Main Effects (c, T, s, t) on M_n_ of PEUs

**Figure 4:**
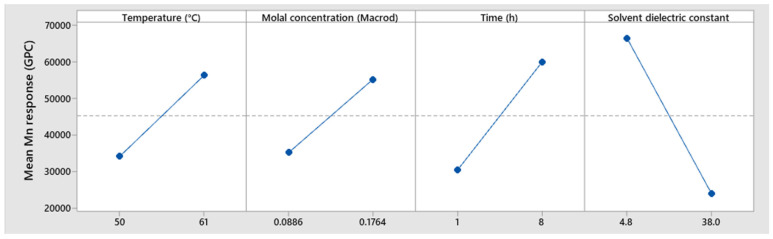
The effect of the main factors of the model derived from the 2^4^ factorial design. Each dot in the graphic illustrates the average of 16 experiments.

To examine the main effects of c, T, s, and t on the *M*_n_ of PEUs, Figure 4 presents a graph that illustrates the impact of each factor at two levels—high and low (as defined in Table 1)—with *M*_n_ values quantified by gel permeation chromatography (GPC). In this graph, the steepness of the slope between the average *M*_n_ values at the two levels provides a measure of the magnitude of the effect for each factor.

The first main effect, moving from left to right in Figure 4, is temperature (T). A change from the low level (50 °C) to the high level (61 °C) results in a substantial increase in the average *M*_n_, rising from 31,350 g/mol to 46,940 g/mol. This represents a 50% increase in *M*_n_ when the temperature is raised from 50 °C to 61 °C.

The second main effect pertains to the initial molal concentration (c) of HOPCLOH. Increasing the concentration from the low level (0.089 molal) to the high level (0.186 molal) results in a significant improvement in the average *M*_n_, rising from 30,140 g/mol to 48,450 g/mol, which corresponds to a 60% increase.

The third main effect is reaction time (t). Extending the reaction duration from 1 h (low level) to 8 h (high level) leads to an increase in the average *M*_n_ from 27,850 g/mol to 51,450 g/mol, representing an 85% improvement.

The fourth main effect is the choice of solvent (*s*). Two solvents with different dielectric constants were used: chloroform (*ε* = 4.8) and acetonitrile (*ε* = 38). The average *M*_n_ values for these solvents were 58,725 g/mol and 22,915 g/mol, respectively. This represents a substantial increase of 150% when switching from acetonitrile to chloroform.

**Figure 5:**
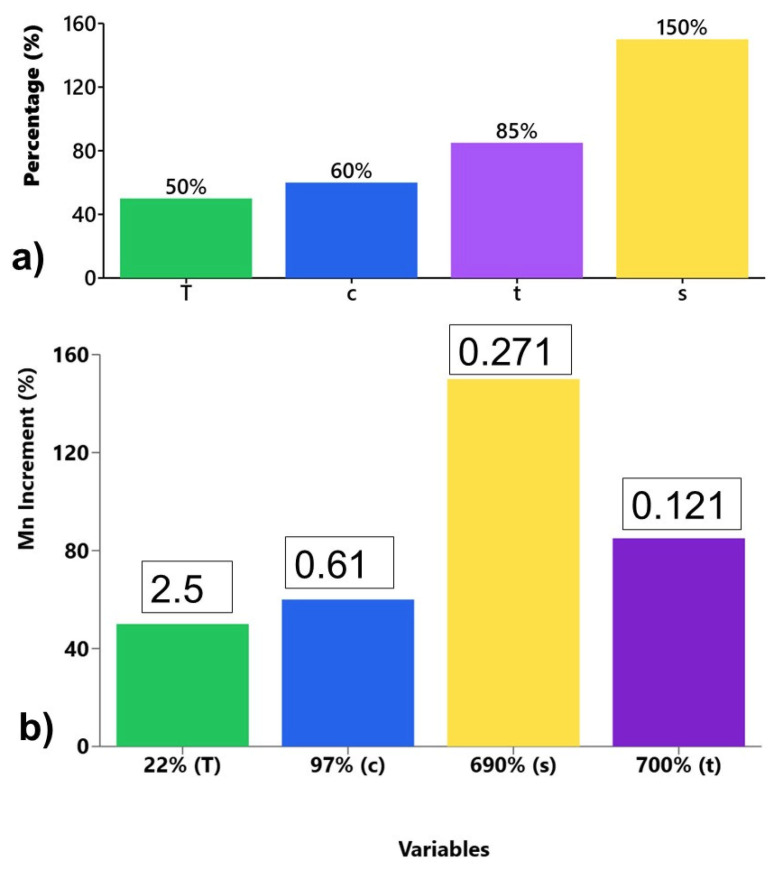
(**a**) The figure presents the percentage change in *M*_n_ (Δ*M*_n_%) corresponding to each primary effect (T, c, t, and s), as determined through the experimental design. (**b**) The figure showcases the *relative impact*, calculated as the ratio of Δ*M*_n_% to the percentage change in each variable (ΔT%, Δc%, Δs%, and Δt%).

Figure 5 provides a clearer perspective on the magnitude of these effects in terms of *M*_n_ increases from low to high levels. In Figure 5a, the percentage increase in *M*_n_ follows the following order: T (50%) < c (60%) < t (85%) < s (150%). However, when analyzing the absolute growth in *M*_n_ (Figure 5b), the ranking changes, with temperature (T) showing the lowest increase, while the effects on *M*_n_ follow the following pattern: T > c > s > t.

To aid in the interpretation of Figure 5b, the relative impact of each variable is defined as follows:

$$\mathrm{Relative}\;\mathrm{Impact}=\frac{∆Mn\%}{∆x\%}$$

where:

- Δ*M*_n_% represents the percentage change in the response variable (*M*_n_) caused by variations in the variables;
- Δ*x*% corresponds to the percentage change in each variable (*T*: temperature, *c*: concentration, *s*: solvent dielectric constant, and *t*: time).

The numerical values within each box in Figure 5b indicate the relative impact (RI) of the respective variable. Among these variables, temperature (T) demonstrates the most pronounced influence on the response variable, with an RI of 2.5. This is followed by concentration (c), with an RI of 0.61, the dielectric constant of the solvent (s) with 0.271, and, finally, reaction time (t), which exhibits the lowest RI at 0.121. These results underscore that, while all factors contribute to changes in *M*_n_, temperature has the greatest relative impact when normalized to the percentage change in the variable.

The solvent was the only factor that failed to meet the assumption of homogeneity of variances. This deviation, however, was not deemed significant enough to compromise the validity of the experimental results. Furthermore, certain combinations of factor levels (interaction effects) were found to elevate the average *M*_n_ above 60,000 g/mol.

**Figure 6:**
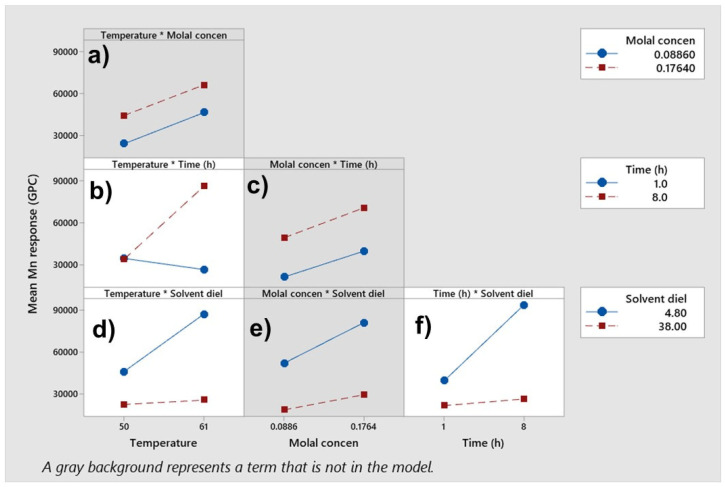
This figure illustrates the significant and non-significant effects of the two-way interactions between the factors in the model derived from the 2^4^ factorial design. (**a**) Temperature and molal concentration (T•c) interaction. (**b**) Temperature and time (T•t) interaction. (**c**) Molal concentration and time (c•t) interaction. (**d**) Temperature and solvent (T•s) interaction. (**e**) Molal concentration and solvent (dielectric constant) (c•s) interaction. (**f**) Time and solvent (dielectric constant) (t•s) interaction.

In Figure 6, each dot in the graphic illustrates the average of eight experiments, and Figure 6d depicts the dual interaction effects, specifically the interaction between temperature and solvent (*T*•*s*). The data reveal that increasing the temperature from 50 °C to 61 °C while using chloroform as the solvent results in a substantial increase in the average *M*_n_, rising from 45,870 g/mol to 87,170 g/mol. In contrast, the same temperature increase with acetonitrile as the solvent produces only a marginal change in *M*_n_, with values increasing from 22,440 g/mol to 25,530 g/mol. Table S3 confirms that the combination of 61 °C and chloroform (ε = 4.8) generates the highest statistically significant average *M*_n_ (87,176 g/mol). Conversely, the two lowest combinations of factor levels involve acetonitrile as the solvent, demonstrating its lower efficacy in achieving high *M*_n_ values under the tested conditions. This evidence further validates temperature as a key factor, as illustrated in Figure 5b.

The temperature and time (T•t) interaction plot is shown in Figure 6b. This graph illustrates that, when the reaction time is 1 h, increasing the temperature from 50 to 61 °C results in only a modest increase in *M*_n_ from 26,491 to 34,502 g/mol. Detailed comparisons of the means for the temperature and time interaction are provided in the Supplementary Material (Table S4). According to Table S4, the highest statistically significant *M*_n_ value (86,220 g/mol) is achieved when the reaction is conducted at 61 °C for 8 h. In contrast, the other level combinations—1 h at 50 °C, 1 h at 61 °C, and 8 h at 50 °C—yield statistically similar *M*_n_ values (34,502, 33,811, and 26,491 g/mol, respectively).

The interaction between reaction time and solvent type is depicted in Figure 6f. The data reveal that, when acetonitrile is used as the solvent, increasing the reaction time from 1 h to 8 h produces only a slight change in *M*_n_ from 21,620 to 26,350 g/mol. However, when chloroform is used as the solvent, the same increase in reaction time leads to a substantial rise in *M*_n_ from 39,370 to 93,670 g/mol, as reported in Table S5. The highest mean *M*_n_ value (93,670 g/mol) is achieved with 8 h of reaction time and chloroform as the solvent. In contrast, the other three mean values (39,370, 26,350, and 21,620 g/mol) are statistically equivalent, highlighting the critical role of the combination of chloroform and extended reaction time in significantly increasing *M*_n_.

Table 3 summarizes the results of the mean comparisons for the three-way interaction between temperature, time, and solvent type. The analysis reveals that most combinations of these factors produce statistically similar *M*_n_ values. However, one combination stands out: a reaction temperature of 61 °C, a reaction time of 8 h, and chloroform (*dielectric constant 4.8*) as the solvent. This specific combination yields a significantly higher *M*_n_ value of 140,910 g/mol.

This analysis confirms that only the specific combination of high temperature, extended reaction time, and chloroform as the solvent results in a substantially higher *M*_n_, while the other combinations do not lead to significant variations in *M*_n_.

Based on the results from the 2^4^ factorial design, the optimal conditions for synthesizing high-*M*_n_ PEUs were identified as follows: a temperature of 61 °C, a reaction time of 8 h, a 0.1876 molal concentration of HOPCLOH, and chloroform (ε = 4.8) as the solvent. These conditions produced the highest *M*_n_ values observed in this study.

Additionally, it was noted that the molal concentration of HOPCLOH is the only factor that does not exhibit any interaction effects. This indicates that changes in *M*_n_ due to concentration are independent of the levels of the other factors. In other words, increasing the concentration level will either significantly or nominally increase *M*_n_, regardless of temperature, reaction time, or solvent type (within the studied reaction space). This behavior is visually supported by the three gray-background graphs in Figure 6a,c,e, where the nearly parallel lines demonstrate that concentration changes proportionally affect the *M*_n_ of PEUs. In contrast, the other factors—temperature, reaction time, and solvent—exhibit various interaction effects, meaning their influence on *M*_n_ depends on the levels of other factors.

The choice of solvent significantly affects the polymerization of HOPCLOH with HDI. Acetonitrile, a polar solvent with a high dielectric constant (*ε* = 38) and low vapor pressure, leads to low conversions of HOPCLOH. Its high polarity likely impedes polymerization by forming strong interactions with hydroxyl (OH) groups, reducing their availability for reaction with HDI. Additionally, during film formation on a Teflon surface, the slow evaporation of acetonitrile facilitates the gradual crystallization of unreacted HOPCLOH, which was detected by a relatively high enthalpy value (Δ*H*_m_). Furthermore, the hygroscopic nature of acetonitrile promotes side reactions between HDI and water, further decreasing polymerization efficiency. However, this side reaction is minimal and only occurs during the evaporation step at room temperature to produce the PEU films. As a result, molecular weight (*M*_n_) values remain consistently low with minimal variation.

In contrast, chloroform, a less polar solvent with higher vapor pressure, improves polymerization efficiency. In a closed system (reflux), it facilitates the interaction between HDI and OH groups, promoting polymerization. In an open system, the rapid evaporation of chloroform inhibits some PCL crystallization, enabling continued polymer growth, including branching and cross-linking. The filtration of PEU samples prior to GPC analysis was notably difficult due to a partially insoluble fraction, likely caused by cross-linking and branching. The insoluble fraction has been previously reported for theoretically linear PUs using an excess of diisocyanate [48,78]. Experimentally, the signal of cross-linking attributed to allophanate is detected at around 1680 cm^−1^, and, usually, at the same wavenumber (cm^−1^), the signal of the urethane group overlaps [77,79]. This result yields higher *M*_n_ values and greater variability compared to acetonitrile.

Polydispersity (*M*_n_/*M*_n_) was analyzed as a complementary response variable in the 2^4^ experimental design. Levene’s test confirmed that chloroform produced significantly greater variability compared to acetonitrile. The primary factors influencing polydispersity were reaction time and solvent choice, with significant three-way interactions also observed. Longer reaction durations, higher temperatures, and the use of chloroform led to increased cross-linking and branching, which broadened the molecular weight distribution (as shown in Table S6). Consequently, while chloroform facilitates higher molecular weights, it also results in increased polydispersity due to enhanced branching and cross-linking during polymerization.

**Figure 7:**
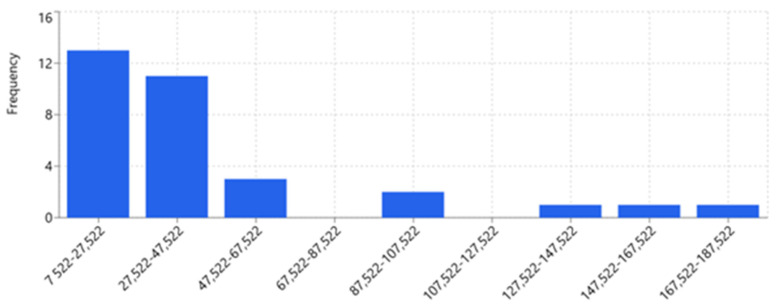
Plot of frequency and range of *M*_n_ of all data from design 2^4^.

On the other hand, the analysis of the frequency vs. *M*_n_ plot, which ideally should resemble a Gaussian distribution, reveals a right skew toward higher *M*_n_ values (Figure 7). The Ryan–Joiner normality test confirmed that the data do not follow a normal distribution, indicating a lack of normality in the *M*_n_ values. This skewness suggests the presence of non-normal behavior in the distribution of *M*_n_, likely influenced by the interaction of the experimental factors.

In Figure 7, it is clear that the highest *M*_n_ values correspond to the last five PEU samples, all synthesized using chloroform as the solvent. The pronounced spacing between these data points indicates that, in addition to yielding higher *M*_n_ values, the use of chloroform introduces greater variability in the *M*_n_ data. This observation is statistically supported by Bartlett’s test for solvent type, which confirms that the variability in *M*_n_ associated with chloroform is significantly greater than that observed with acetonitrile.

## 5. Contrasting *M*_n_ (GPC) of PEUs with Other Response Variables

### 5.1. OH/ESTER Response by ^1^H NMR

The number-average molecular weight (*M*_n_) of poly(ester-urethanes) (PEUs) can also be estimated using ^1^H NMR spectroscopy by analyzing the OH/ESTER response. This metric reflects the ratio of signal intensities corresponding to hydroxyl (OH) and ester groups, providing insights into the conversion of unreacted HOPCLOH in the final PEU product (see Supporting Information Table S7). By combining conversion data from the OH/ESTER response with the known *M*_n_ values of 1,6-hexamethylene diisocyanate (HDI) and HOPCLOH, the *M*_n_ of the synthesized PEUs can be calculated (Table S7).

However, as the proportion of unreacted HOPCLOH decreases with increasing polymerization, the precision of *M*_n_ determined via ^1^H NMR diminishes. This is due to integration errors associated with low-intensity signals in the NMR spectrum, particularly when *M*_n_ values are high. Therefore, caution is required when interpreting *M*_n_ values for PEUs with high degrees of polymerization, as these estimates are inherently less reliable.

**Figure 8:**
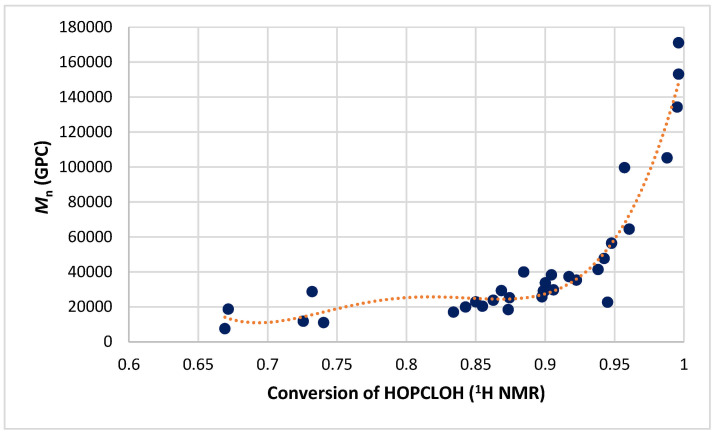
A graph depicting the *M*_n_, as determined by gel permeation chromatography (GPC), plotted against the conversion of HOPCLOH, as quantified through hydrogen nuclear magnetic resonance (^1^H NMR) analysis (OH/ESTER response).

Figure 8 demonstrates the relationship between *M*_n_ of PEUs, as determined by GPC, and HOPCLOH conversion calculated from the OH/ESTER response. The data exhibit a typical step-growth polymerization behavior, where *M*_n_ remains low for conversions below 0.95, but rises sharply once conversion exceeds this threshold. This aligns with the characteristic mechanism of step-growth polymerization, where high molecular weights are achieved only at near-complete monomer conversion. It is important to note that the OH/ESTER response is not the only way to contrast *M*_n_ values obtained via GPC. Other NMR-based metrics, such as the OH/URET response, demonstrate similar behavior and trends (see Section S1 of the Supporting Information).

### 5.2. Enthalpy of Fusion (ΔH_m_) Response by DSC

**Figure 9:**
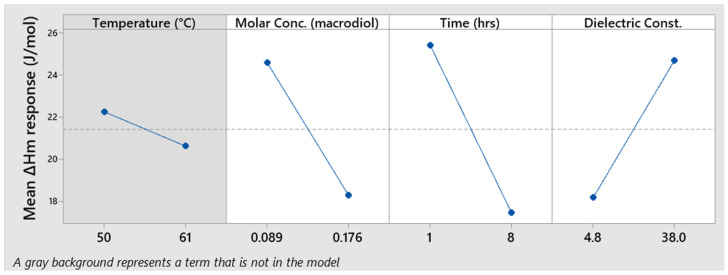
Main effects plot of the response Δ*H*_m_ with each of the factors initially considered in the 2^4^ factorial design model. Each dot in the graphic illustrates the average of 16 experiments.

All PEU samples were analyzed via differential scanning calorimetry (DSC). In a typical DSC thermogram, the primary thermal transition observed was the melting temperature (*T*_m_) and its associated enthalpy of fusion (Δ*H*_m_). Since all PEU samples exhibited a *T*_m_, the entire series of PEUs can be classified as semicrystalline polymers. The Δ*H*_m_ was attributed to the polycaprolactone (PCL) soft segment within the PEUs. An inverse correlation was observed between *M*_n_(GPC) and Δ*H*_m_. The 2^4^ factorial design revealed that all main factors, except temperature, significantly influenced Δ*H*_m_ and *T*_m_, while no significant interaction effects were detected (Figure 9). Conditions that reduced Δ*H*_m_ correlated with those that maximized *M*_n_. At high *M*_n_, the increased concentration of urethane groups promotes hydrogen bonding, which inhibits the crystallization of some PCL segments. Furthermore, chemical cross-linking at high *M*_n_ further suppresses crystallinity. The optimal conditions for minimizing crystallinity and melting temperature were identified as a reaction time of 8 h, the use of chloroform as the solvent (ε = 4.8), and a 0.176 molal concentration of HOPCLOH. These conditions align with those that produce the highest *M*_n_, further supporting the inverse relationship between *M*_n_ and Δ*H*_m_.

## 6. Characterization of PEUs by Mechanical Properties

**Figure 10:**
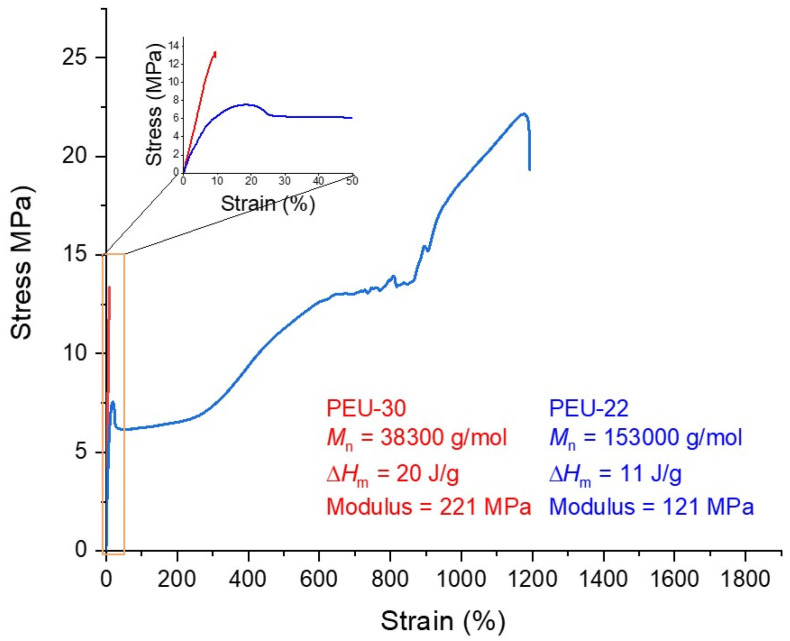
The stress–strain curves of two PEU samples. The samples were synthesized using chloroform as solvent, but they differ in other synthesis factors. The red curve represents sample PEU-30 (50 °C, 0.089 molal, 8 h), and the blue curve represents sample PEU-22 (61 °C, 0.176 molal, 8 h).

Mechanical testing was performed on a subset of PEUs synthesized as part of the 2^4^ factorial design. However, many films were excluded from testing due to their fragility. Notably, all PEUs with *M*_n_ values above 64,000 g/mol, exclusively synthesized using chloroform as the solvent, exhibited elongations exceeding 1000%. In contrast, samples with lower *M*_n_ values and those synthesized using acetonitrile exhibited intermediate-to-low elongations. Figure 10 presents the stress–strain curve for a representative PEU-22 (similar to PEU-13, Figure 1) synthesized with chloroform. This sample displayed a high *M*_n_ (≈153,000 g/mol), an elastic modulus of 121 MPa, a Δ*H*_m_ of 11 J/g, and a significantly high elongation at break (≈1200%). The curve demonstrated classic plastic behavior, consistent with findings in previous publications [23,25,37,71]. In contrast, a typical sample synthesized using acetonitrile as the solvent (PEU-20) exhibited poor mechanical properties due to its low molecular weight and fragile physical appearance, indicative of a relatively low *M*_n_. These results underscore the critical role of solvent choice in determining the mechanical and structural properties of PEUs. Specifically, chloroform facilitates the synthesis of higher-molecular-weight polymers, resulting in improved elongation at break and enhanced mechanical performance compared to acetonitrile.

The mechanical properties of PEU-30 (*M*_n_ ≈ 38,000 g/mol) and PEU-22 (*M*_n_ ≈ 153,000 g/mol) are compared in Figure 10 (stress–strain curves), where the influence of *M*_n_ on the mechanical behavior of PEUs is evident. Both samples were synthesized using chloroform as the solvent. PEU-30 exhibited a high modulus (221 MPa) compared to PEU-22. This is attributed to the relatively high enthalpy of the fusion (Δ*H*_m_ = 20 J/g) of the PCL soft segment in PEU-30, which contributes to increased stiffness but results in limited elongation at break (approximately 10%), leading to poor mechanical performance.

In contrast, PEU-22 demonstrated a significantly higher elongation at break, consistent with its high *M*_n_ and the presence of repetitive urethane units that promote intermolecular hydrogen bonding. Additionally, covalent cross-linking, likely involving allophanate groups, may be present in PEU-22. This cross-linking enhances cohesion between polymer chains, allowing the material to withstand greater elongation before fracturing. These findings highlight the critical role of molecular weight and cross-linking density in governing the mechanical properties of PEUs. While higher *M*_n_ and cross-linking contribute to improved elongation and toughness, in contrast, increased crystallinity, as observed in PEU-30, leads to enhanced stiffness but reduced ductility.

## 7. Conclusions

α,ω-Hydroxy telechelic poly(ε-caprolactone) (HOPCLOH) was synthesized through the ring-opening polymerization (ROP) of ε-caprolactone (CL). HOPCLOH was obtained with a low degree of polymerization (DP = 10) as it served as the starting material for the synthesis of poly(ester-urethanes) (PEUs) in the presence of 1,6-hexamethylene diisocyanate (HDI). The chemical structures of both HOPCLOH and PEUs were validated using nuclear magnetic resonance (NMR), differential scanning calorimetry (DSC), and polarized optical microscopy (POM).

A 2^4^ statistical factorial design was systematically employed to prepare 32 PEU samples, where four main factors—concentration (c), temperature (T), solvent (s), and reaction time (t)—were investigated to optimize the number-average molecular weight (*M*_n_), as determined by gel permeation chromatography (GPC), as the primary response. The results indicated significant double and triple interactions between the main factors. The optimal conditions for maximizing *M*_n_ were identified as a concentration of 0.176 molal, a temperature of 61 °C, a reaction time of 8 h, and chloroform as the solvent (dielectric constant: 4.8). Among the factors, temperature exhibited the most significant influence on *M*_n_ compared to concentration, solvent, and reaction time.

The choice of solvent in a polyaddition mechanism, such as PEU synthesis, was shown to be critical. Acetonitrile yielded the lowest *M*_n_ values, while chloroform provided the most favorable environment for chain propagation, resulting in significantly higher *M*_n_ values. Additionally, NMR analysis revealed that the OH/ESTER response was inversely proportional to *M*_n_ (GPC).

The *M*_n_ of PEUs was found to have a pronounced impact on their thermal and mechanical properties, with most samples exhibiting a semicrystalline structure and plastic mechanical behavior. These findings suggest that future research could focus on exploring a broader range of solvents to identify additional options for optimizing PEU synthesis. A more comprehensive study of solvent effects could provide valuable insights into improving the efficiency and scalability of PEU production.

## Figures and Tables

**Table 1:** The four factors are shown with their two levels of 2^4^ factorial design.

**Levels**	**Factors**				**Total Experiments 32**
	**Temperature (°C)**	**HOPCLOH Concentration** **(Molality)**	**Reaction Time (h)**	**Solvent Type (Dielectric Constant)**	
**High level**	61	0.176	8	Acetonitrile (38)	
**Low level**	50	0.089	1	Chloroform (4.8)	

**Table 2:** The significant main factors and interactions considered in the model derived from the original 2^4^ factorial design.

No	Source	DF	SS Adj.	MS Adj.	F Value	*p* Value
1	Temp (°C)	1	3,942,342,609	3,942,342,609	16.06	0.00055243
2	Molal conc. (molality)	1	3,198,620,149	3,198,620,149	13.03	0.00147501
3	Time (h)	1	697,075,220	697,075,220	28.39	2.0721 × 10^−5^
4	Dielectric const. (Solvent)	1	1.4474 × 10^10^	1.4474 × 10^10^	58.96	8.6 × 10^−8^
5	Temp (°C) × Time (h)	1	7300760075	7,300,760,075	29.74	1.5261 × 10^−5^
6	Temp (°C) × Dielectric const. (Solvent)	1	2,920,485,845	2,920,485,845	11.9	0.00218247
7	Time (h) × Dielectric const. (Solvent)	1	4,912,709,345	4,912,709,345	20.01	0.00017298
8	Temp (°C) × Time (h) × Dielectric const. (Solvent)	1	4,217,591,285	4,217,591,285	17.18	0.00039241
	Error	23	5,646,470,439	245,498,715		
	Lack of fit	7	1,270,754,470	181,536,353	0.66	0.69928137
	Pure error	16	4,375,715,970	273,482,248		
	Total	31	5.3584 × 10^10^			

Notes: DF: Degrees of Freedom. SS Adj.: Adjusted Sum of Squares. MS Adj.: Adjusted Mean Square.

**Table 3:** Summary of comparisons of means for the interaction between temperature, time, and solvent type (*M*_n_ response by GPC).

Temperature × Time × Type of Solvent (°C, Hours, Dielectric Constant)	N	Mean	Group
61, 8, 4.8	4	140,910	A
50, 8, 4.8	4	46,430	B
50, 1, 4.8	4	45,300	B
61, 1, 4.8	4	33,440	B
61, 8, 38.0	4	31,530	B
50, 1, 38.0	4	23,690	B
50, 8, 38.0	4	21,180	B
61, 1, 38.0	4	19,540	B

Means that do not share a letter are significantly different.

## Data Availability

The original contributions presented in this study are included in the article/Supplementary Material. Further inquiries can be directed to the corresponding authors.

## Supplementary Materials

The following supporting information can be downloaded at: https://www.mdpi.com/article/10.3390/polym17050668/s1, Scheme S1. Synthesis of poly(ε-caprolactone) from 1,8-octanediol and ε-caprolactone, using ammonium decamolybdate as a catalyst; Figure S1: It shows the FT-IR spectrum of the urea sample derived from HDI and water; Figure S2. Variable OH/URET response vs. the two levels of: temperature, concentration, time, and type of solvent (dielectric constant); Figure S3. 1H NMR spectrum of acetonitrile used as a solvent in the synthesis of PEUs from the 2^4^ factorial design; Figure S4. This figure illustrates the two-way interaction effects incorporated into the model derived from the 2^4^ factorial design. Among the evaluated factors, temperature and dielectric constant emerged as the only significant effects. The analysis was conducted using the OH/URET response; Table S1. Each possible combination of factor levels (treatments) of the design is shown 2^4^; Table S2. All combinations of factors (reaction temperature and time, molar concentration of the macrodiol, and type of solvent) from the experimental design 24 are presented. The results of two response variables, specifically polydispersity and the number-average molecular weight (Mn), are shown. Additionally, the relative ambient humidity percentage during the synthesis of polyurethanes is reported; Table S3. Summary of the comparison of means for the interaction Temperature*Type of solvent (*M*_n_ response by GPC); Table S4. Summary of comparisons of means of the interaction Temperature*Time; Table S5. Summary of comparison of means Time*Type of solvent; Table S6. The comparison of various levels within the three-way interaction among temperature, solvent type (characterized by dielectric constant), and reaction time—based on the model developed from a 2^4^ factorial design—has been summarized, focusing on the polydispersity response; Table S7. Calculation of Mₙ by ¹H NMR from the quantification of HOPCLOH (OH/ESTER response) before and after its reaction with the diisocyanate; Table S8. The Analysis of Variance (ANOVA) for the 24 experimental design is presented, using the average molecular weight (Mn) obtained via Gel Permeation Chromatography (GPC) as the response variable. Effects that were determined to be statistically significant are highlighted in bold and italics; Table S9. Factors influencing the different response variables (response Mn, OH/ESTER response, ΔH_m_ response, and OH/URET response); Table S10. Presents the proposed rangea of band assignments in the FT-IR spectrum for the polyurethane (PEUs) synthesized in this study; Table S11. The results of the response variables ΔHm (J/g) and Tm (°C) are shown; Table S12. Simplified ANOVA table derived from the 2^4^ factorial design using the ESTER/URET response; Table S13. This table presents a summary of the comparison of significant three-way interactions identified using the OH/URET response. These interactions were derived from the model based on the 2^4^ factorial design; Table S14. It shows the reduced ANOVA derived from the model of 2^4^ factorial design using the polydispersity response.
