# Supplementary material for: Experimental Design (24) to Improve the Reaction Conditions of Non-Segmented Poly(ester-urethanes) (PEUs) Derived from α,ω-Hydroxy Telechelic Poly(ε-caprolactone) (HOPCLOH)
Source: Polymers (Basel). 2025 Feb 28;17(5):668. doi: 10.3390/polym17050668 (PMC11902744; doi:10.3390/polym17050668)
Supplement: Supplementary file 1 [file polymers-17-00668-s001.zip › polymers-3483279-supplementary.pdf]

Supplementary Information

Experimental design (2^4) to improve the reaction conditions of non-segmented poly(ester-urethanes) (PEUs) derived from α,ω-hydroxy telechelic poly(ε-caprolactone) (HOPCLOH)

Jaime Maldonado-Estudillo [1], Rodrigo Navarro-Crespo [2], Ángel Marcos-Fernández,[2] María Dolores de Dios Caputto [2], Gustavo Cruz-Jiménez [3], José E. Báez [1,*]

[1] Department of Chemistry, University of Guanajuato, Noria Alta S/N Guanajuato, Gto. 36050 México. Correspondence: jebaez@ugto.mx (J.E.B.)

[2] Instituto de Ciencia y Tecnología de Polímeros (ICTP), Consejo Superior de Investigaciones Científicas (CSIC), C/Juan de la Cierva No. 3, 28006, Madrid, Spain.

[3] Department of Pharmacy, University of Guanajuato, Noria Alta S/N Guanajuato, Gto. 36050 Mexico.

Table S1 Each possible combination of factor levels (treatments) of the design is shown 2^4

Repetition		Temperature	Concentration	Time	Solvent polarity
A	B	-1	-1	-1	-1
A	B	1	-1	-1	-1
A	B	-1	1	-1	-1
A	B	1	1	-1	-1
A	B	-1	-1	1	-1
A	B	1	-1	1	-1
A	B	-1	1	1	-1
A	B	1	1	1	-1
A	B	-1	-1	-1	1
A	B	1	-1	-1	1
A	B	-1	1	-1	1
A	B	1	1	-1	1
A	B	-1	-1	1	1
A	B	1	-1	1	1
A	B	-1	1	1	1
A	B	1	1	1	1

Note: The numbers 1 and -1 are the high and low levels respectively of each factor. Each treatment was performed twice (two replicates) and randomized.

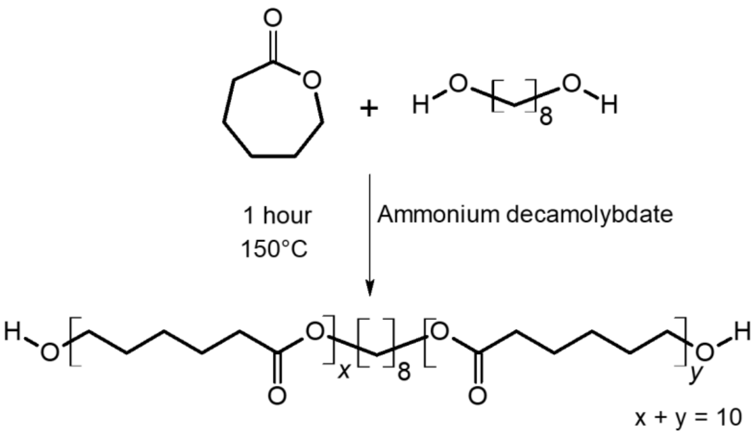

Scheme S1. Synthesis of poly(ε-caprolactone) from 1,8-octanediol and ε-caprolactone, using ammonium decamolybdate as a catalyst.

Table S2 All combinations of factors (reaction temperature and time, molar concentration of the macrodiol, and type of solvent) from the experimental design 2^4 are presented. The results of two response variables, specifically polydispersity and the number-average molecular weight (M_n), are shown. Additionally, the relative ambient humidity percentage during the synthesis of polyurethanes is reported.

Run	Sample	Temp (°C)	Molar Conc. Macrodiol	Time (h)	Dielectric Const. Solvent	Polydispersity (GPC)	M_n (GPC) (g/mol)	Relative Humidity (%)
1	PEU-1	61	0.1764	8	38	1.79	41316	57.0
2	PEU-2	61	0.0886	8	4.8	2.83	134291	43.0
3	PEU-3	61	0.1764	1	4.8	2.03	56370	30.0
4	PEU-4	50	0.1764	1	38	2.01	39930	29.5
5	PEU-5	61	0.0886	8	4.8	2.64	105220	54.5
6	PEU-6	61	0.0886	1	38	1.91	29094	43.0
7	PEU-7	50	0.0886	8	38	1.89	22841	37.5
8	PEU-8	61	0.1764	8	38	1.77	37268	52.0
9	PEU-9	61	0.1764	1	38	1.95	20344	57.0
10	PEU-10	50	0.0886	1	4.8	1.97	29283	59.0
11	PEU-11	50	0.1764	8	4.8	2.11	64499	60.0
12	PEU-12	50	0.1764	8	38	1.65	25170	59.0
13	PEU-13	61	0.1764	8	4.8	5.15	171051	65.0
14	PEU-14	61	0.1764	1	38	1.74	16970	49.5
15	PEU-15	50	0.0886	8	4.8	1.81	35274	67.0
16	PEU-16	61	0.0886	1	4.8	1.86	19912	58.0
17	PEU-17	61	0.0886	8	38	1.96	18384	61.5
18	PEU-18	50	0.1764	8	4.8	1.9	47662	63.0
19	PEU-19	50	0.1764	1	4.8	1.91	22646	63.5
20	PEU-20	50	0.0886	1	38	1.62	7522	57.0
21	PEU-21	61	0.0886	1	4.8	1.69	23798	77.0
22	PEU-22	61	0.1764	8	4.8	3.36	153083	41.0
23	PEU-23	61	0.0886	8	38	2.03	29142	35.5
24	PEU-24	50	0.1764	1	38	1.78	28687	62.5
25	PEU-25	50	0.0886	1	4.8	2.02	29672	42.5
26	PEU-26	50	0.0886	8	38	2.01	10982	67.5
27	PEU-27	50	0.1764	8	38	1.94	25754	47.5
28	PEU-28	61	0.0886	1	38	1.61	11758	57.0
29	PEU-29	50	0.1764	1	4.8	3.93	99625	56.0
30	PEU-30	50	0.0886	8	4.8	2.07	38308	52.5
31	PEU-31	61	0.1764	1	4.8	1.71	33688	47.5
32	PEU-32	50	0.0886	1	38	1.7	18651	54.0

Note: A statistical correlation between M_n and environmental relative humidity (%) was sought but found to be insignificant.

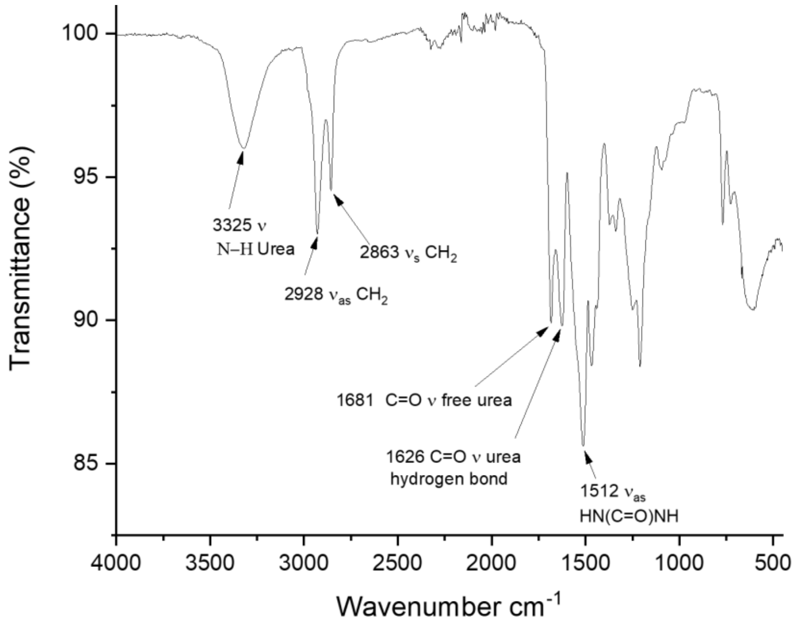

Figure S1. It shows the FT-IR spectrum of the urea sample derived from HDI and water.

The data were initially fitted to a multiple linear regression model (15 effects were considered).

Equation S1*:* $M_n = \beta_0 + \beta_1 T + \beta_2 c + \beta_3 s + \beta_4 t + \beta_5 Tc + \beta_6 Ts + \beta_7 Tt + \beta_8 cs + \beta_9 ct + \beta_{10} st + \beta_{11} Tcs + \beta_{12} Tct + \beta_{13} Tst + \beta_{14} cst + \beta_{15} Tcst + \varepsilon$

Where:

T: Temperature

c: concentration

s: solvent

t: time

M_n: Dependent variable

β_0: Intercept

β_1 to β_{15}: Regression coefficients

ε: Random error

Terms comprising multiple letters (e.g., Ts, ct) denote interaction effects between the respective variables.

After the first ANOVA, only 8 effects were determined to be significant:

Equation S2*:* $M_n = \beta_0 + \beta_1 T + \beta_2 c + \beta_3 s + \beta_4 t + \beta_6 Ts + \beta_7 Tt + \beta_{10} st + \beta_{13} Tst + \varepsilon$

Table S3. Summary of the comparison of means for the interaction Temperature*Type of solvent (M_n response by GPC).

Temperature*Type of Solvent	N	Mean (g/mol)	Group
61°C, Chloroform	8	87 176.6	A
50°C, Chloroform	8	45 871.1	B
61°C, Acetonitrile	8	25 534.5	B, C
50°C, Acetonitrile	8	22 442.1	C

Means that do not share a letter are significantly different.

Table S4. Summary of comparisons of means of the interaction Temperature*Time.

Temperature*Time (°C, hours)	N	Mean (g/mol)	Group
61, 8	8	86 219.4	A
50, 1	8	34 502.0	B
50, 8	8	33 811.3	B
61, 1	8	26 491.8	B

Means that do not share a letter are significantly different.

Table S5. *Summary of comparison of means Time*Type of solvent.*

**Time*Type of solvent (hours, dielectric constant)**	**N**	**Mean (g/mol)**	**Group**
8, 4.8	8	93 673.5	A
1, 4.8	8	39 374.3	B
8, 38.0	8	26 357.1	B
1, 38.0	8	21 619.5	B

Means that do not share a letter are significantly different.

Table S6. The comparison of various levels within the three-way interaction among temperature, solvent type (characterized by dielectric constant), and reaction time—based on the model developed from a 2^4 factorial design—has been summarized, focusing on the polydispersity response.

Temp (hrs)*Solvent Const(°C)*Dielec. Time	Number of samples	Mean of the transformed polydispersity response	Group
61 8 4.8	4	3.12	A
50 1 4.8	4	2.14	A
50 8 4.8	4	1.96	B
61 8 38.0	4	1.88	B
50 8 38.0	4	1.86	B
61 1 4.8	4	1.81	B
61 1 38.0	4	1.78	B
50 1 38.0	4	1.76	B

The means that do not share a letter are significantly different.

Table S7. Calculation of *M*[illegible] by ^{1}H NMR from the quantification of HOPCLOH (OH/ESTER response) before and after its reaction with the diisocyanate

Sample	HOPCLOH after reacting	HOPCLOH before / HOPCLOH after	Conversion	Degree of polymerization	*M*[illegible] PEU from NMR (g/mol)	*M*[illegible] (GPC) (g/mol)
PEU-1	0.0127	0.0619	0.9381	16.16	20776	41316
PEU-2	0.001	0.0049	0.9951	205.30	263852	134291
PEU-3	0.0107	0.0521	0.9479	19.18	24659	56370
PEU-4	0.0237	0.1154	0.8846	8.66	11133	39930
PEU-5	0.0025	0.0122	0.9878	82.12	105541	105220
PEU-6	0.0208	0.1013	0.8987	9.87	12685	29094
PEU-7	0.0308	0.1500	0.8500	6.66	8567	22841
PEU-8	0.017	0.0828	0.9172	12.07	15521	37268
PEU-9	0.0298	0.1452	0.8548	6.88	8854	20344
PEU-10	0.027	0.1315	0.8685	7.60	9772	29283
PEU-11	0.0081	0.0395	0.9605	25.34	32574	64499
PEU-12	0.0258	0.1257	0.8743	7.95	10227	25170
PEU-13	0.0008	0.0039	0.9961	256.62	329814	171051
PEU-14	0.0341	0.1661	0.8339	6.02	7738	16970
PEU-15	0.0159	0.0774	0.9226	12.91	16594	35274
PEU-16	0.0323	0.1573	0.8427	6.35	8169	19912
PEU-17	0.026	0.1266	0.8734	7.89	10148	18384
PEU-18	0.0118	0.0575	0.9425	17.39	22360	47662
PEU-19	0.0113	0.0550	0.9450	18.16	23350	22646
PEU-20	0.0679	0.3307	0.6693	3.02	3886	7522
PEU-21	0.0282	0.1374	0.8626	7.28	9356	23798
PEU-22	0.0008	0.0039	0.9961	256.62	329814	153083
PEU-23	0.0206	0.1003	0.8997	9.96	12808	29142
PEU-24	0.055	0.2679	0.7321	3.73	4797	28687
PEU-25	0.0193	0.0940	0.9060	10.63	13671	29672
PEU-26	0.0533	0.2596	0.7404	3.85	4950	10982
PEU-27	0.021	0.1023	0.8977	9.77	12564	25754
PEU-28	0.0563	0.2742	0.7258	3.64	4687	11758
PEU-29	0.0088	0.0429	0.9571	23.32	29983	99625
PEU-30	0.0196	0.0955	0.9045	10.47	13462	38308
PEU-31	0.0205	0.0999	0.9001	10.01	12871	33688
PEU-32	0.0674	0.3283	0.6717	3.04	3915	18651

Note: The OH/ESTER response (HOPCLOH before) before reacting is 0.1823

From the OH/ESTER response before and after the reaction, it is possible to determine the degree of polymerization (DP). The OH/ESTER response before the

reaction corresponds to 100% of the hydroxyl groups that are potentially going to react with the diisocyanate groups. Similarly, the same response quantifies the hydroxyl groups present after polymerization in the PEU films. In other words, this response measures the amount of hydroxyl groups (OH) before and after the reaction.

Based on this, ***M***□ was calculated from the degree of polymerization.

Initially, the degree of conversion was calculated:

$$x = \frac{N_0 - N}{N_0}$$

Where:

- $\mathbf{N_0}$ is the OH/ESTER signal response before polymerization.
- **N** is the OH/ESTER signal response after polymerization of HOPCLOH with HDI.

Subsequently, once a degree of conversion (**x**) was obtained, the degree of polymerization was determined:

$$DP = \frac{1}{1 - x}$$

Finally, using the degree of polymerization (**DP**) and the average-molecular weight obtained by ^{1}H NMR of HOPCLOH ($M_n(RMN)_{HOPCLOH}$) and the molecular weight of HDI ($M_{w_{HDI}}$), ***M***□ **(RMN)** for all PEU samples was calculated:

$$M_{\mathrm{n}}\ (\mathrm{RMN}) = GP(M_n(RMN)_{HOPCLOH} + M_w HDI)$$

S1 section.

^{1}H NMR responses

In the 2^4 factorial design, the relative quantity of unreacted HOPCLOH (denoted as the OH/URET response) in the final PEU was quantified. All main factors—temperature, concentration, reaction time, and solvent type—significantly influenced the OH/URET response, while no statistically significant interaction effects were observed. A negative correlation between the OH/URET ratio and M_n was confirmed: a lower OH/URET ratio corresponded to a higher M_n (see **Figure S2**). Optimized conditions for minimizing the OH/URET response were identified as 61°C, a molality of 0.176, a reaction time of 8 hours, and the use of chloroform (dielectric constant = 4.8) as the solvent. These conditions reduce hydroxyl group content and increase urethane group concentration in the PEU sample.

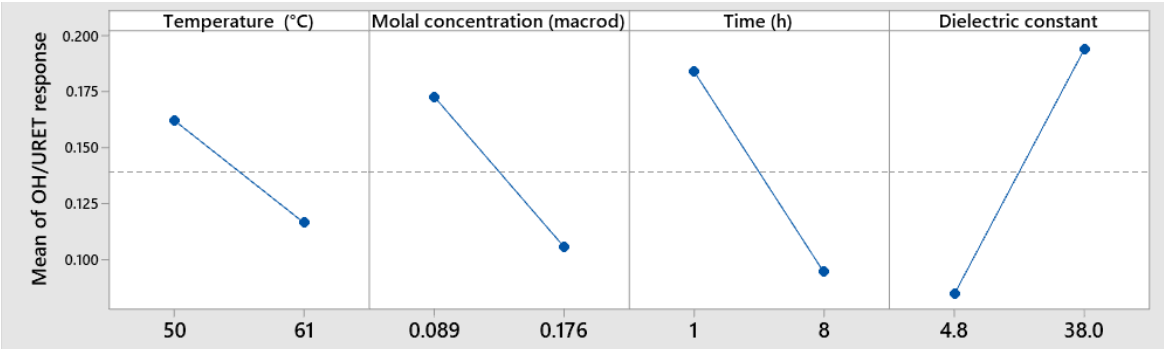

Figure S2. Variable OH/URET response vs. the two levels of: temperature, concentration, time, and type of solvent (dielectric constant).

Similarly, the OH/ESTER response was included as a response variable in the same experimental design. As with the OH/URET response, all main factors were significant, with no interaction effects detected. Moreover, the optimal conditions identified for minimizing the OH/URET response were consistent with those for the OH/ESTER response and other variables in this study.

Table S8. The Analysis of Variance (ANOVA) for the 2^4 experimental design is presented, using the average molecular weight (M_n) obtained via Gel Permeation Chromatography (GPC) as the response variable. Effects that were determined to be statistically significant are highlighted in bold and italics.

Source	DF	SS Adj.	MS Adj.	F-Value	p-Value
Model	15	4.920E+10	3,280,544,108	12.00	0.000005
Linear	4	2.858E+10	7,146,465,149	26.13	0.000001
Temp (°C)	***1***	***3942342609***	***3,942,342,609***	***14.42***	***0.001584***
Macrod Conc	***1***	***3198620149***	***3,198,620,149***	***11.70***	***0.003509***
Time (hrs)	***1***	***6970705220***	***6,970,705,220***	***25.49***	***0.000119***
Solvent Dielec. Const	***1***	***1.447E+10***	***1.447E+10***	***52.93***	***0.000002***
2-way Interactions	6	1.581E+10	2,635,794,929	9.64	0.000143
Temp (°C)*Macrod Conc	1	271769	271,769	0.001	0.975242
Temp (°C)*Time (hrs)	***1***	***7300760075***	***7,300,760,075***	***26.70***	***0.000094***
Temp (°C)*Solvent Dielec. Const	***1***	***2920485845***	***2,920,485,845***	***10.68***	***0.004836***
Macrod Conc*Time (hrs)	1	16232178	16,232,178	0.059	0.810617
Macrod Conc*Solvent Dielec. Const	1	664310363	664,310,363	2.43	0.138661
Time (hrs)*Solvent Dielec. Const	***1***	***4912709345***	***4,912,709,345***	***17.96***	***0.000626***
3-way Interactions	4	4807236342	1,201,809,086	4.39	0.013789
Temp (°C)**Macrod Conc**Time (hrs)	1	472404638	472,404,638	1.73	0.207278
Temp (°C)**Macrod Conc**Solvent Dielec. Const	1	116666631	116,666,631	0.43	0.522939
Temp (°C)*Time (hrs)*Solvent Dielec. Const	***1***	***4217591285***	***4,217,591,285***	***15.42***	***0.001203***
Macrom Conc*Time (hrs)*Solvent Dielec. Const	1	573788	573,788	0.0021	0.964033
4-way Interactions	1	295104	295,104	0.0011	0.974201
Temp (°C)**MacromConc**Time (hrs)*Solvent Dielec. Const	1	295104	295,104	0.0011	0.974201
Error	16	4375715970	273,482,248		

Table S9. Factors influencing the different response variables (response M_n, OH/ESTER response, ΔH_m response, and OH/URET response)

Response Variable	Favored by	Disfavored by
Response M_n	Increase in T, c, t	Increase in solvent dielectric constant
OH/ESTER response (amount of precursor in PEUs)	Increase in solvent dielectric constant	Increase in T, c, t
OH/URET response	Increase in solvent dielectric constant	Increase in T, c, t
ΔH_m of the soft segment in PEU (ΔH_m response)	Increase in solvent dielectric constant	Increase in T, c, t
Mechanical properties	Increase in T, c, t	Increase in solvent dielectric constant

Legend:

- T: Temperature
- c: Concentration
- t: Time

Table S10. Presents the proposed range[a] of band assignments in the FT-IR spectrum for the polyurethane (PEUs) synthesized in this study.

Group	Mode	Frequency (cm^{-1})
[-O-<u>C</u>O-$(CH_2)_5$-] (ester)	C=O······H-N	1704 - 1713
[-O-<u>C</u>O-$(CH_2)_5$-] (ester)	Free	1716 - 1726
[(-CH_2-NH-<u>CO</u>-NH-$)_2$], urea	C=O······H-N (ordered)	1620 - 1635
[(-CH_2-NH-<u>CO</u>-NH-$)_2$], urea	Free	1690 - 1700
[(-CH_2-NH-<u>CO</u>-O-$)_2$], urethane	C=O······H-N	1680 - 1690
[(-CH_2-NH-<u>CO</u>-O-$)_2$], urethane	Free	1710 - 1720
[(-CH_2-<u>NH</u>-CO-O-$)_2$], urethane	N-H······N-H and N-H······O=C (ester)	3330 - 3390
[(-CH_2-<u>NH</u>-CO-O-$)_2$], urethane	Free	3445 - 3450

[a]Assignment of the range of infrared spectra signals based on Yilgör's table. Yilgör E, Wilkes GL. Critical parameters in designing segmented polyurethanes and their effect on morphology and properties: A comprehensive review. Polymer. 2015 Feb 10;58: A1-36. Doi: https://doi.org/10.1016/j.polymer.2014.12.014

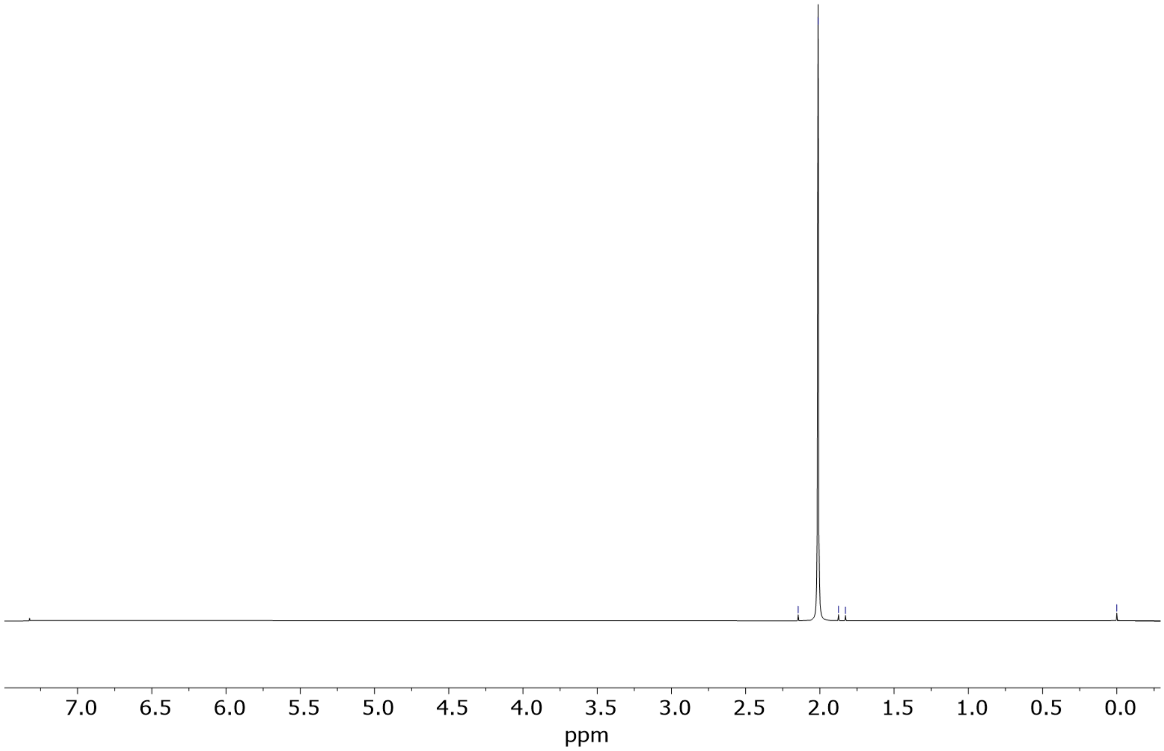

Figure S3. ^{1}H NMR spectrum of acetonitrile used as a solvent in the synthesis of PEUs from the 2^4 factorial design.

Note: No traces of moisture were detected in the acetonitrile spectrum.

Table S11. The results of the response variables ΔH_m (J/g) and T_m (°C) are shown.

Run Order	Temperature (°C)	Molar Conc. (macrodiol)	Time (hrs)	Dielectric Const.	Response ΔH_m (J/g)
1	61	0.1764	8	38	13.64
2	61	0.0886	8	4.8	15.58
3	61	0.1764	1	4.8	13.81
4	50	0.1764	1	38	17.66
5	61	0.0886	8	4.8	12.12
6	61	0.0886	1	38	28.43
7	50	0.0886	8	38	25.03
8	61	0.1764	8	38	16.91
9	61	0.1764	1	38	25.76
10	50	0.0886	1	4.8	26.92
11	50	0.1764	8	4.8	10.61
12	50	0.1764	8	38	18.86
13	61	0.1764	8	4.8	10.84
14	61	0.1764	1	38	25.65
15	50	0.0886	8	4.8	20.3
16	61	0.0886	1	4.8	31.42

17	61	0.0886	8	38	18.84
18	50	0.1764	8	4.8	14.31
19	50	0.1764	1	4.8	24.23
20	50	0.0886	1	38	31.4
21	61	0.0886	1	4.8	28.08
22	61	0.1764	8	4.8	11.42
23	61	0.0886	8	38	19.63
24	50	0.1764	1	38	35.26
25	50	0.0886	1	4.8	16.42
26	50	0.0886	8	38	34.08
27	50	0.1764	8	38	17.9
28	61	0.0886	1	38	38.49
29	50	0.1764	1	4.8	16.51
30	50	0.0886	8	4.8	18.93
31	61	0.1764	1	4.8	18.96
32	50	0.0886	1	38	27.34

Table S12. Simplified ANOVA table derived from the 2^4 factorial design using the ESTER/URET response.

Source	DF	SS Adj.	MS Adj.	F Value	p Value
Temperature (°C)	1	0.19065	0.19065	8.49	0.0078332
Molal concentration (molality)	1	0.34653	0.34653	15.42	0.0006741
Time (hrs)	1	0.86133	0.86133	38.33	0.0000026
Dielectric constant	1	0.43478	0.43478	19.35	0.0002083
Temperature (°C) * Time (hrs)	1	0.0639	0.0639	2.84	0.1052349
Temperature (°C) * Dielectric constant	1	0.12625	0.12625	5.62	0.0265309
Time (hrs) * Dielectric constant	1	0.04728	0.04728	2.1	0.160406
Temperature (°C) * Time (hrs) * Dielectric constant	1	0.114	0.114	5.07	0.0341359
Error	23	0.5168	0.02247		
Lack of fit	7	0.16945	0.02421	1.12	0.4006128
Pure error	16	0.34735	0.02171		
Total	31	2.70152			

Notes:

DF: Degrees of freedom

SS Adj.: Adjusted sum of squares

MS Adj.: Adjusted mean square

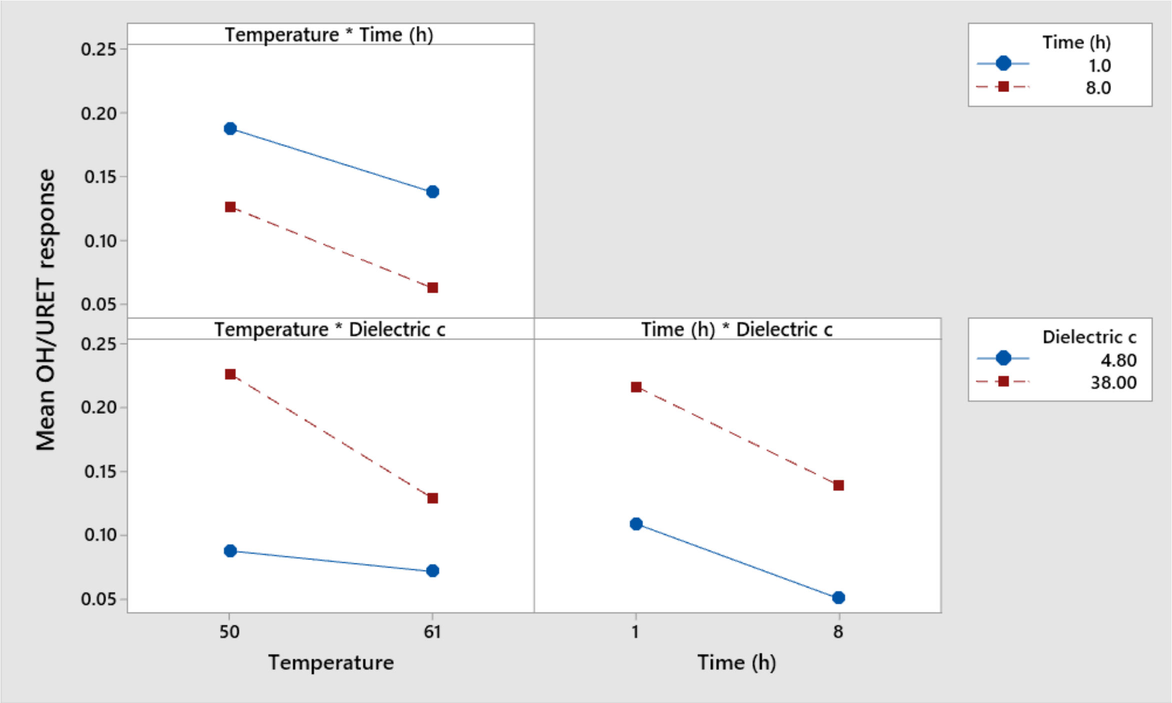

Figure S4. This figure illustrates the two-way interaction effects incorporated into the model derived from the 2^4 factorial design. Among the evaluated factors, temperature and dielectric constant emerged as the only significant effects. The analysis was conducted using the OH/URET response

Table S13. This table presents a summary of the comparison of significant three-way interactions identified using the OH/URET response. These interactions were derived from the model based on the 2^4 factorial design.

**Temperature (°C)*Time (h)*Dielectric constant**	**Number of observations**	**Mean of OH/URET response**	**Group**
50°C* 1h* 38.0	4	0.2825	A
50°C* 8°C* 38.0	4	0.17	B
61°C* 1h* 38.0	4	0.15	B
61°C* 1h* 4.8	4	0.125	B, C
61°C* 8h* 38.0	4	0.1075	B, C
50°C* 1h* 4.8	4	0.0925	B, C
50°C* 8h* 4.8	4	0.0825	B, C
61°C* 8h* 4.8	4	0.0175	C

Note: Means that do not share a letter are significantly different.

Table S14. It shows the reduced ANOVA derived from the model of 2^4 factorial design using the polydispersity response.

**Source**	**GL**	**SC Ajust.**	**MC Ajust.**	**Value F**	**Value p**
Temp (°C)	1	0.03753	0.037531	0.63	0.437
Time (hrs)	1	0.43559	0.435586	7.26	0.013
Const dielec. Solvent	1	0.95357	0.953574	15.9	0.001
Temp (°C)*Time (hrs)	1	0.48914	0.489144	8.15	0.009
Temp (°C)*Const dielec. Solvent	1	0.00798	0.007982	0.13	0.718
Tiempo (hrs)*Const dielec. Solvent	1	0.04906	0.049061	0.82	0.375
Temp (°C)*Time(hrs)*Const dielec. Solvent	1	0.50899	0.508992	8.48	0.008
Error	24	1.43979	0.059991		
Lack of adjustment	8	0.48838	0.061048	1.03	0.456
Error pure	16	0.9514	0.059463		
Total	31	3.92166			
